# Shaping beta diversity in arid landscape through native plant species contributions: synergy of climate, soil, and species traits

**DOI:** 10.3389/fpls.2025.1521596

**Published:** 2025-03-10

**Authors:** Reham Fekry El-Barougy, Louis-Félix Bersier, Sarah M. Gray, Ali El-Keblawy, Tarek Galal, Fazal Ullah, Ibrahim A. Elgamal, Mohammed A. Dakhil

**Affiliations:** ^1^ Botany and Microbiology Department, Faculty of Science, Damietta University, Damietta, Egypt; ^2^ Department of Biology - Ecology and Evolution, University of Fribourg, Fribourg, Switzerland; ^3^ Department of Biological Sciences, University of Toronto Scarborough, Toronto, ON, Canada; ^4^ Department of Applied Biology, Faculty of Science, University of Sharjah, Sharjah, United Arab Emirates; ^5^ Department of Biology, College of Sciences, Taif University, Taif, Saudi Arabia; ^6^ State Key Laboratory of Grassland Agro-ecosystems, College of Ecology, Lanzhou University, Lanzhou, China; ^7^ Nature Conservation Sector, Egyptian Environmental Affairs Agency, Cairo, Egypt; ^8^ School of Ecology and Environment, Northwestern Polytechnical University, Xi’an, China; ^9^ Botany and Microbiology Department, Faculty of Science, Helwan University, Cairo, Egypt

**Keywords:** climatic aridity, species turnover, phylogenetic diversity, species traits, soil resources, species abundance

## Abstract

Understanding how species traits, climate aridity, and soil resources interact to influence beta diversity is critical for predicting changes in plant community composition. This study aims to investigate how these interactions shape species contributions to spatial turnover and beta diversity, focusing on the unique dryland ecosystems of the Saint Katherine Protectorate (SKP) in Egypt. To address this, we analyzed data from 84 vegetation plots, considering the direct and indirect effects of climatic aridity, soil resources, and species traits (e.g., plant height, leaf production, specific leaf area), as well as the relative abundance of C3 plants and phylogenetic diversity on species contribution to beta diversity (SCBDeff). Using Generalized Linear Models (GLMs) and Structural Equation Modelling (SEMs), the results revealed complex indirect effects of aridity and soil resources on SCBDeff mediated by plant traits. SCBDeff was positively influenced by climatic aridity, particularly in species with greater phylogenetic distance, taller plants, high leaf production, and a higher relative abundance of C3 plants. Conversely, specific leaf area (SLA) had a negative effect. Phylogenetic diversity emerged as a significant driver of beta diversity, with distantly related species contributing more due to functional differentiation and niche partitioning. The findings emphasize the critical role of species traits and environmental conditions in shaping beta diversity. These insights can inform conservation strategies aimed at enhancing ecosystem stability under shifting climatic conditions, particularly in dryland environments where species adaptive traits play a pivotal role.

## Introduction

Understanding the factors influencing the spatial distribution of species diversity is fundamental in ecology, as it forms the basis for biodiversity conservation and ecosystem management (MacArthur and Wilson, 1967; Hubbell, 2001; Chave, 2013). Beta diversity, defined as the variation in species composition among communities, encompasses two primary components; species turnover, which identifies regions with distinct ecological communities, and nestedness, which reflects differences in species richness due to gain or loss of species across sites (Whittaker, 1960; Legendre and De Cáceres, 2013; Baselga, 2010). Mapping the spatial variation of beta diversity identifies areas with high species turnover or unique assemblages, supporting the conservation of ecosystems vulnerable to environmental stressors like those in arid regions (Legendre and De Cáceres, 2013; Soininen et al., 2018; Frasconi Wendt et al., 2021). These arid regions, known for their unique species assemblages and high turnover rates, are primary conservation foci due to increased risks from climate change and habitat fragmentation (Nagendra et al., 2013; Maestre et al., 2012; Sala et al., 2000). These regions also face additional challenges such as habitat degradation and desertification, which are exacerbated by global change (Reynolds et al., 2007).

Legendre and De Cáceres (2013) proposed the partitioning of beta diversity into Local Contributions to Beta Diversity (LCBD) and Species Contributions to Beta Diversity (SCBDeff), which allows ecologists to discern the roles of spatial factors, species traits, and abiotic drivers (e.g., climate, soil resources) in shaping biodiversity patterns (Legendre and Gauthier, 2014; Chiu et al., 2014). Local Contributions to Beta Diversity (LCBD), on the other hand, identifies ecologically distinct sites that are essential for regional biodiversity conservation, particularly in regions vulnerable to habitat loss and degradation (Legendre and Gauthier, 2014; Cadotte and Tucker, 2017). SCBDeff pinpoints key species driving community composition differences, highlighting those that play significant ecological roles and are crucial for ecosystem stability (Anderson et al., 2011; Villéger et al., 2013). Species with high SCBDeff values are vital for ecosystem functionality, and their decline could trigger notable biodiversity loss, making their conservation imperative. This is particularly critical in arid areas, where environmental stressors such as water scarcity and extreme temperatures amplify the vulnerability of ecosystems to biodiversity loss (De et al., 2023; Liu et al., 2024; Mouillot et al., 2013). Recognizing these species is instrumental in developing conservation actions aimed at maintaining ecosystem resilience and function (Pillar et al., 2013; Soininen et al., 2018; Gonzalez et al., 2020).

A comprehensive understanding of beta diversity requires not only an examination of species distributions but also an exploration of the evolutionary relationships among these species. Phylogenetic diversity plays a critical role in explaining beta diversity and species turnover by providing insights into the evolutionary relationships among species within communities. Closely related species often exhibit similar ecological niches due to shared ancestry, which can lead to lower beta diversity as these species tend to co-occur in similar environments (Webb et al., 2002; Cavender-Bares et al., 2009). Conversely, distantly related species, which have diverged more significantly in evolutionary terms, contribute to higher beta diversity by occupying distinct niches and adapting to different environmental conditions (Graham and Fine, 2008; Swenson, 2011). This phylogenetic divergence often results in greater species turnover across environmental gradients, as different lineages respond to various selective pressures (Hardy and Senterre, 2007). By integrating phylogenetic information, researchers can better understand how evolutionary history influences current patterns of beta diversity, offering deeper insights into the mechanisms driving community assembly and ecosystem functioning (Tucker et al., 2017). This understanding lays the groundwork for examining how both evolutionary and ecological processes interact to shape biodiversity patterns, thereby complementing studies on species-specific traits and environmental gradients.

Examining SCBDeff typically involves interactions between environmental and species-based characteristics, such as occupancy, abundance, niche breadth, and niche position. These traits can exhibit inter-correlation (Tales et al., 2004; Heino, 2005; Siqueira et al., 2009; Heino and Grönroos, 2017). For instance, species with narrow niche breadths may inhabit constrained environments, contributing significantly to beta diversity (Brown, 1984; Slatyer et al., 2013). Similarly, species in marginal habitats often exist in restricted environments, impacting SCBDeff differently from species in non-marginal areas (Doledec et al., 2000; Heino and Grönroos, 2014). Investigating the biotic and abiotic factors that influence SCBDeff is also essential (Legendre and De Cáceres, 2013; Anderson et al., 2011) as it allows to pinpoint species that significantly influence compositional dissimilarities between communities, providing insights into species-specific roles in ecological processes (Baselga, 2010; Podani and Schmera, 2011; Socolar et al., 2016). These insights help predict how environmental conditions, species traits, or community interactions may alter community composition and ecosystem functioning (Legendre and De Cáceres, 2013). SCBDeff enhances the detection of keystone or functionally distinct species that disproportionately shape community structure, biodiversity patterns, and ecosystem resilience (Anderson et al., 2011). Despite the extensive focus on LCBD, SCBDeff studies are relatively limited, particularly in how environmental stressors and species traits influence beta diversity (Rodríguez-Lozano et al., 2023).

Traits that influence species’ ability to adapt to environmental gradients and ecological pressures were chosen for their key role in shaping beta diversity and co-occurrence patterns. Species traits such as seed mass and dispersal capacity were known due to their well-documented influence on beta diversity by shaping species occupancy and abundance patterns (Verberk et al., 2010; Heino and Grönroos, 2014). While many traits could impact beta diversity and co-occurrence patterns, this study focuses on traits that are particularly relevant in structuring communities across environmental gradients. Traits like drought resistance, water use efficiency, and nutrient uptake strategies are especially crucial in stress-prone ecosystems, enabling species to endure harsh conditions while maintaining functional diversity and ecosystem resilience (Chaturvedi et al., 2021). Plant functional traits such as specific leaf area (SLA), leaf nitrogen content, and wood density are emphasized due to their role in influencing resource acquisition strategies and spatial turnover. For instance, SLA is associated with dominance in resource-rich environments, contributing to spatial variation and beta diversity (Violle et al., 2007). By focusing on these traits, this study aims to highlight their role in shaping community structure and adaptation to stressors like aridity, thereby influencing beta diversity (Suding et al., 2008; Díaz et al., 2016).

This study investigated the direct, indirect, and interactive effects of abiotic factors including climatic factors and soil factors, and biotic factors including species traits on species contributions to beta diversity (SCBDeff). Our research uniquely integrates climatic aridity, soil resources, and plant traits such as height, specific leaf area (SLA), number of leaves, and the relative abundances of C3 and C4 plants. This multifaceted approach allows us to quantify and characterize the levels of aridity and soil resources comprehensively, shedding light on their direct and indirect effects on SCBDeff. Moreover, the study employs advanced statistical techniques, including structural equation modeling (SEM), to disentangle the complex pathways through which these environmental factors impact SCBDeff via plant traits. These innovative methodologies and the integration of detailed plant traits and environmental variables contribute new insights into how beta diversity operates in arid, mountainous landscapes, providing a foundation for improved conservation strategies in similar ecosystems globally.

## Materials and methods

### Study area

The Saint Katherine Protectorate (SKP) is an arid protected area and a biodiversity hotspot located in south Sinai, Egypt. The SKP diverse geomorphological and geological structures have led to the emergence of various microhabitat types, each harboring distinct ecological niches (Shaltout, 2018). Among these, the Wadis, acting as drainage systems, play a crucial role in water collection and provide favorable conditions for plant growth. However, the persistence of such species-rich ecosystems in this challenging arid landscape remains an intriguing subject of study (Hegazy and Doust, 2016). This region has an arid climate characterized by scarce and unpredictable rainfall, with a mean annual rainfall of about 60 mm. However, the high peaks receive orographic precipitation, some in snow, which may reach up to 300 mm annually. The area is a part of the igneous crystalline Pre-Cambrian formation, which is more than 600 million years old.

The diversity in geomorphological and geological structures of SKP resulted in a unique landscape. Six landform microhabitat types are identified: Wadis (valleys), Terraces, Slopes, Gorges, Cliffs, Farsh (basins), and Caves. Wadis are one of the most important ecosystems in SKP, acting as drainage systems that collect water from catchment areas and form favorable habitats for plant growth (Khedr, 2007; Omar, 2012). These areas are also rich in cultural and natural heritage sites, such as Mount Sinai and the Monastery of St. Catherine, as well as the Wadi Feiran watershed area. SKP harbors a large number of endemic species, further adding to its ecological importance.

### Vegetation sampling

Field surveys were conducted across 84 randomly distributed 20 m × 20 m plots (see **Supplementary Figure S1**), to capture the vegetation composition and species abundance within the region’s sparse vegetation. In each plot, both species presence and abundance were recorded, focusing exclusively on native species. The abundance data was then used to calculate the relative abundance of C3 and C4 plants in the study area. Relative abundance was determined by dividing the total abundance of each plant species by the total abundance of C3 or C4 species within each plot.

In addition to species presence and abundance, we recorded two key ecological characteristics; species dominance, quantified as the percentage cover of each species within plots (%) and life-form categories that were classified into shrubs, herbs, and trees following Raunkiær’s system (Raunkiær, 1934).

We focused on vascular plants, which are the primary contributors to vegetation structure in the area. Surveys were conducted during April–June 2021 to coincide with peak vegetative activity, ensuring that only species with visible vegetative parts were included in the survey. This timing minimizes seasonal biases linked to extreme heat or drought-induced dormancy. This approach ensures that both common and less abundant species were adequately represented, providing a full picture of the vegetation structure across different microhabitats.

### Environmental data and species traits data

The climate data, including the aridity index (AI), were downloaded from the CGIAR-CSI Global database with a resolution of 30 arc seconds (www.cgiar-csi.org, Trabucco et al., 2008; Fisher et al., 2011). These data represent average climate conditions for the period 1970–2000, aligning closely with the vegetation data collected during April–June 2021 to ensure temporal consistency.

The physical and chemical soil properties were represented by five quantitative variables downloaded from the ISRIC-World Soil Information database at a depth of 0–2 m and a spatial resolution of 30 arc seconds. These data reflect soil conditions up to 2022. We used the spatial analyst toolbox in ArcGIS 10.5 to generate mean raster layers for different soil depths, which were then resampled to a 2.5 arc-min (~5 km) resolution. A composite variable representing the five soil variables was computed using Principal Component Analysis (PCA), with the first axis capturing 79% of the variability. This composite variable included soil organic carbon content, soil pH, soil texture, cation exchange capacity, and water availability.

To link site-level environmental variables to species-level analyses, we calculated the mean aridity and soil resource values across all plots where each species occurred. This aggregation transformed site-specific environmental data into species-specific predictors, enabling direct integration with trait-based models of species contributions to beta diversity (SCBDeff). For example, a species occurring in 10 plots was assigned the mean aridity and soil values of those 10 plots, ensuring environmental drivers were contextualized to its realized niche. This approach follows established methods for scaling site-level abiotic factors to species-level responses in heterogeneous landscapes (Araújo et al., 2019).

To address potential multicollinearity among the environmental variables, we performed a Variance Inflation Factor (VIF) analysis using the ‘usdm’ package (version 1.1-18) in R 4.1.1 (Naimi, 2015). A VIF threshold of 10 was applied, which is a commonly used cut-off to identify and exclude variables that exhibit significant multicollinearity. Variables with VIF values above this threshold were excluded from the analysis to ensure robust statistical modeling. The final variables retained after the VIF analysis were the aridity index (AI), soil composite variable (captured through PCA).

The measured plant traits included height from ground level (cm), specific leaf area (SLA) in cm²/g, and the number of leaves per plant (leaf production). Due to restrictions on plant removal, SLA was measured following the method described by El-Barougy et al. (2021a, 2021c). Specific leaf area was estimated using allometric equations (Basuki et al., 2009). We scanned the leaves of native species outside their protected range and measured the total leaf area using the IMAGEJ software, version 1.49. Then, we dried the leaves and determined the leaf dry weight and calculated the SLA (cm2/g) as the leaf area divided by the leaf weight (Basuki et al., 2009).

To evaluate phylogenetic relationships among the native species, a phylogeny was constructed using four commonly sequenced genes available in GenBank (Benson et al., 2012): rbcL, matK, ITS1, and 5.8s. Among the 67 native species, 60 had at least one gene represented in GenBank. For the seven native species without available sequence data, we used sequences from congeneric relatives as a proxy, following phylogenetic guidelines by Cadotte and Jin (2014). Specifically, we selected the closest relatives within the same genus or, when unavailable, within the same family, ensuring that these substitutes shared similar ecological and morphological characteristics with the target species.

Additionally, to establish the root of the phylogeny, the genetic sequence of *Amborella trichopoda* Baill, an early diverging angiosperm, was included as an outgroup species. The phylogenetic tree (see **Supplementary Figure S2**) was constructed using methods previously described in El-Barougy et al. (2021b). Phylogenetic relatedness within plots was calculated using the mean pairwise phylogenetic distance (MPD) following Swenson’s method (2014) with the “MPD” function in the R package picante (version 1.8, Kembel et al., 2010).

### Assessment of species contribution to beta diversity (SCBDeff)

According to Legendre and De Cáceres (2013), we calculated total beta diversity (BDtotal) and species contribution to beta diversity (SCBDeff). BDtotal provides a measure of the overall variability in species composition across all plots within a study. To compute BDtotal, species composition data were first transformed using the Hellinger method (Legendre and Gallagher, 2001), which standardizes the data while preserving ecological distance. This transformation enabled the calculation of SCBDeff, representing each species’ contribution to beta diversity within the dataset. We employed a species abundance matrix, where rows represented species and columns denoted different spatial units, to calculate SCBDeff using the “beta.div” function from the adespatial package in R. SCBDeff was expressed as each species’ percentage contribution, calculated as a fraction of the total contribution.

### Beta regression for calculating SCBDeff

**Figure 1:**
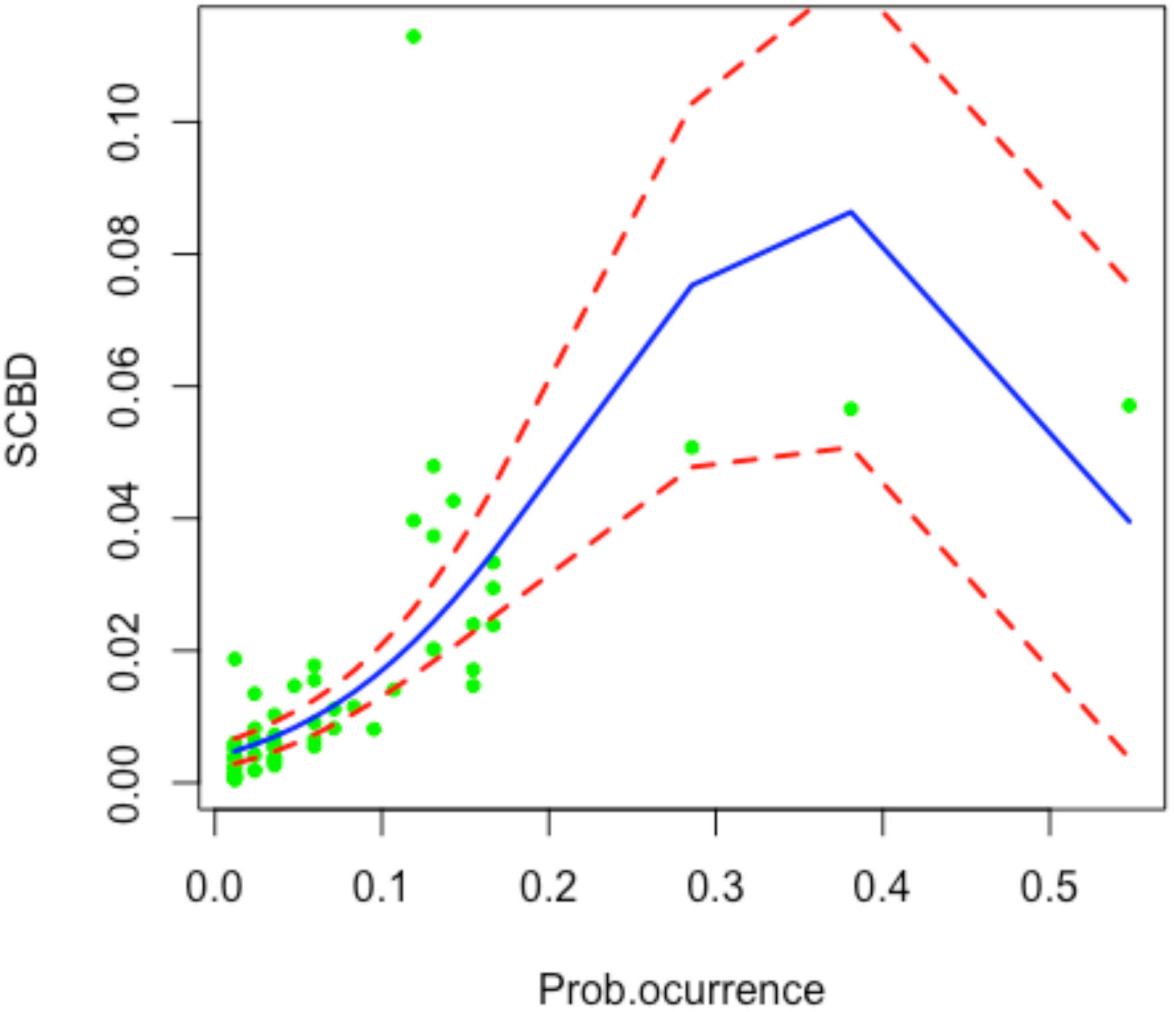
Beta regression illustrating the response of species contributions to beta diversity (SCBD) to the probability of species occurrence. The x-axis represents the probability of species occurrence, while the y-axis shows SCBD values. The solid line indicates the regression trend, and dashed lines represent the 95% confidence intervals.

Since SCBDeff values range between 0 and 1, we applied beta regression, a statistical approach suitable for modeling dependent variables constrained within this interval. This method assumes that the dependent variable follows a beta distribution and relates it to explanatory variables using a linear predictor combined with a logit link function (e.g. Cribari-Neto and Zeileis, 2010). However, a limitation of SCBD analyses is that this metric is inherently influenced by the distribution and abundance of species across sites. Specifically, species that occur only at a single site or ubiquitously across all sites contribute minimally to beta diversity. The contribution of a species to beta diversity is not solely determined by its occurrence frequency but rather by the variability in its abundance across sites and the environmental heterogeneity influencing its distribution (see Heino and Grönroos, 2017; Xia et al., 2022). Consequently, we suggest a two steps approach. First, we remove the effect of occurrence using a quadratic beta regression with occurrence as independent variable (**Figure 1**) by extracting the residuals. We call this metric *effective SCBD* (SCBD_eff_), which is defined for species *i* as: SCBD_eff,_ *_i_* = SCBD*_i_* – predicted (SCBD*_i_*). Second, we use linear models to relate SCBD_eff_ to biologically meaningful explanatory variables (e.g., species traits). For the Beta regression, we utilized the R function “betareg” from the R packages betareg (Cribari-Neto and Zeileis, 2010). This approach aligns with methods for site contribution to beta diversity as proposed by Legendre and De Cáceres (2013).

### Generalized linear models

To explore the influence of aridity and soil resources on SCBDeff, we conducted generalized linear models (GLMs) with Gaussian family distribution, setting SCBDeff as the response variable. Explanatory variables included the soil resources composite, climate aridity index, mean pairwise phylogenetic distance (MPD), the C3/C4 relative abundance ratio, and species-level traits as defined above. Residuals were visually examined using QQ plots, confirming no transformations were necessary. The importance of interaction terms observed in initial models led us to employ structural equation modeling for a detailed analysis of direct and indirect influences.

### Structural equation model

Structural equation modeling (SEM) was utilized to estimate both the direct and indirect effects of explanatory variables on SCBDeff (Grace and Pugesek, 1997; Grace, 2006). We initially constructed a comprehensive model that accounted for all possible direct and indirect effects of the variables under consideration. This model was subsequently refined through backward elimination, guided by the Akaike Information Criterion corrected for small sample sizes (AICc) values.

Prior to model fitting, the distributional assumptions of normality for all variables were assessed using the Shapiro-Wilk test and by examining Q-Q plots. SEM analyses were performed using the lavaan package (version 0.6-9; Rosseel, 2012) in R, with the function “sem” for model fitting and lavaan.survey package (version 1.1.3.1; Oberski, 2014) to account for survey design effects using the lavaan.survey function.

Model adequacy was evaluated using three fit indices: The Standardized Root Mean Square Residual (SRMR), with a threshold of less than 0.08 indicating a good fit; the Goodness-of-Fit Index (GFI), where values greater than 0.95 suggest a good fit; and the chi-squared test, where a P-value greater than 0.05 is considered satisfactory fit (Kenny et al., 2015). These indices were used synergistically rather than hierarchically to provide a comprehensive assessment of model fit, ensuring robust conclusions about model adequacy. All statistical analyses were conducted in R version 4.2.1 (R Core Team, 2022).

## Results

### Influences of the interactions of species traits, climate and soil on SCBD

**Figure 2:**
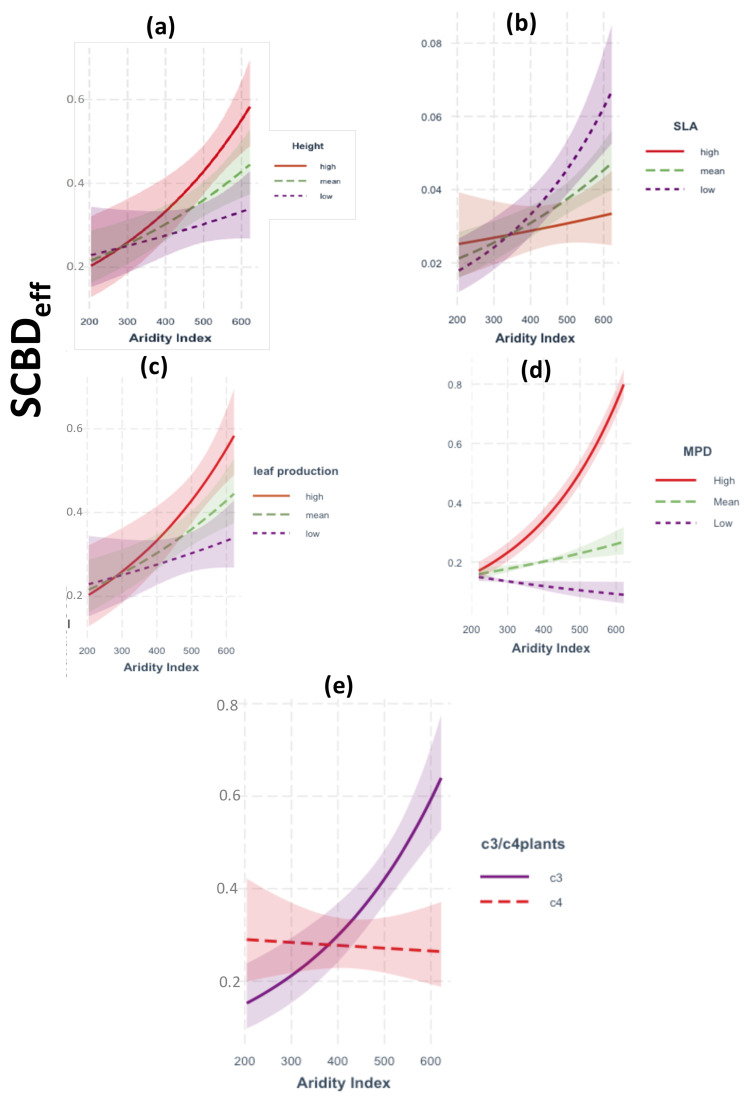
Results of GLM models showing the response of SCBDeff to the two-way interactions between climatic aridity index and: **(a–c)** species traits, **(d)** phylogenetic relatedness (MPD), **(e)** C3 vs C4 plant species. In panels a to d, red solid line (High) represents species in the upper 75th percentile of the trait distribution; green dashed line (Mean) between the 25th and the 75th percentile, and purple dotted line (Low) in the lower 25th percentile. Shaded regions: Indicate the 95% confidence intervals around the regression lines.

**Figure 3:**
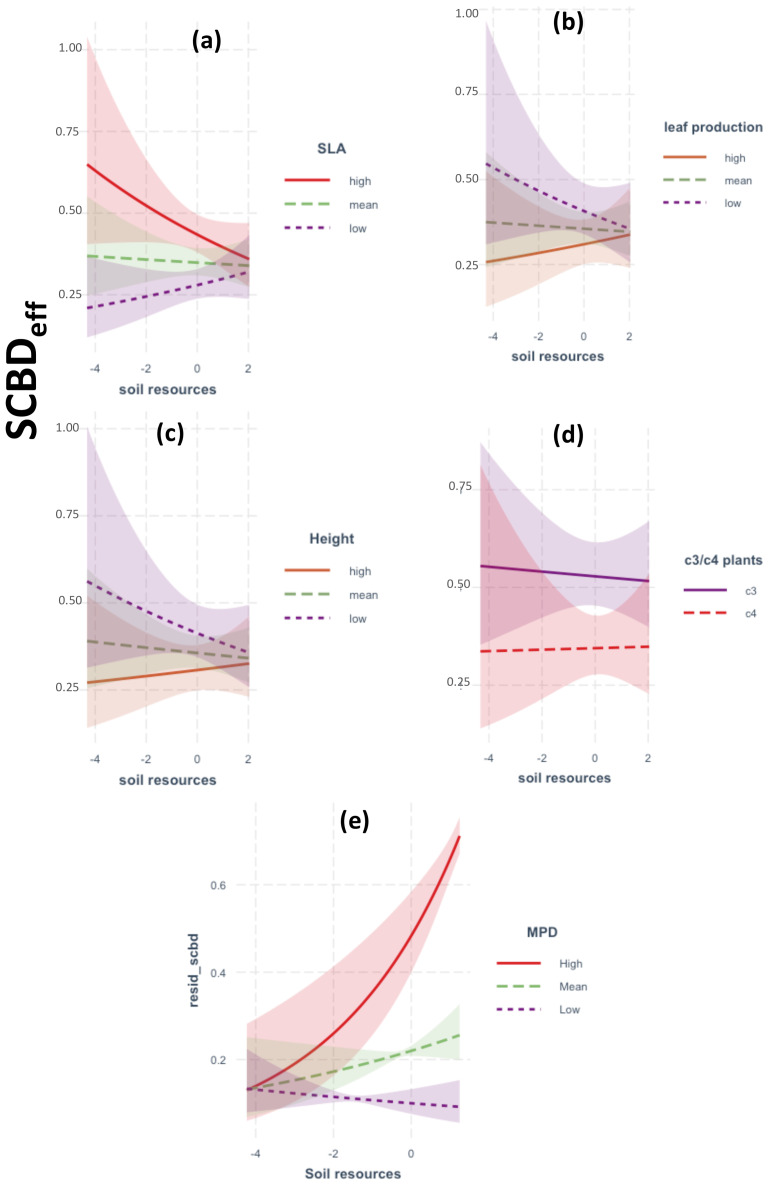
Results of GLM models showing the response of SCBDeff to the two-way interactions between soil resources and: **(a–c)** species traits, **(d)** C3 vs C4 plant species, **(e)** phylogenetic relatedness (MPD). Legend as in **Figure 2**.

A total of 67 native plant species was recorded during the survey period (April–June 2021; see **Supplementary Table S1**). The GLM analysis of two-way interactions demonstrated that SCBDeff was significantly influenced by climatic aridity and soil resources, with notable modulation by species traits (**Figures 2**, **3**). Specifically, SCBDeff showed a positive and significant interaction with climatic aridity (β = 0.002, p = 0.001), which was further intensified by high values of mean phylogenetic distance (MPD), the relative abundance of C3 plants, increased plant height, and high leaf production, while low specific leaf area (SLA) also contributed positively (**Figure 2**). In contrast, SCBDeff exhibited a negative interaction with soil resources (β = -0.262, p = 0.001), with the direction and strength of this negative effect strongly influenced by low SLA values alongside high plant height and leaf production (**Figure 3**). These findings warranted the inclusion of species traits in the SEM analysis to examine their direct and indirect interactions with climatic aridity and soil resources.

### Direct and indirect effects on SCBD

**Figure 4:**
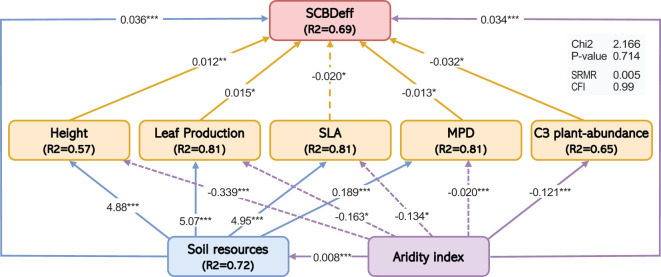
Structural equation model showing the effects of the climatic aridity index, soil resources, and species traits on SCBDeff. Numbers adjacent to arrows are path coefficients. Solid and dashed arrows represent positive and negative relationships, respectively. Only significant pathways are shown (P < 0.05). Asterisks (*) indicate the significance levels of the path coefficients (*P < 0.05, **P < 0.01, ***P < 0.001). The proportion of variance explained (R²) appears alongside each response variable in the model. The goodness-of-fit statistics (Chi2 and associated P-value, SRMR, CFI) are presented in the gray table within the model.

The SEM results provided comprehensive insights into the direct and indirect influences of species traits and their interactions with climate and soil predictors on SCBDeff. SCBDeff was significantly and positively associated with plant height, leaf production, and the relative abundance of C3 plants (β = 0.01, 0.01, 0.032; p = 0.01, 0.026, 0.01, respectively), suggesting that increases in leaf production and the relative abundance of C3 plants are directly linked to higher contributions to beta diversity among native species. Conversely, SLA exhibited a significant direct negative effect on SCBDeff (β = -0.02, p = 0.027), indicating that species with high SLA values tend to have lower contributions to beta diversity. Additionally, MPD had a significant direct effect on SCBDeff, indicating that distantly related species contribute more to beta diversity than closely related species (**Table 1**; **Figure 4**).

**Table 1:** Results of the SEM for the direct and indirect effects of the climatic aridity index, soil resources, and species traits on SCBDeff.

Response Variable	Predictors	Coefficient	Std. Err	Z-value	P(>|z|)
Direct Effects					
SCBDeff	Height (cm)	0.012	0.002	5.309	0.001
	SLA	-0.02	0.013	-1.523	0.027
	Leaf Production	0.015	0.004	2.232	0.026
	MPD	0.013	0.034	0.383	0.02
	Aridity Index	0.034	0.005	6.328	0.000
	Soil resources	0.036	0.006	6.328	0.000
	C3 plants-abundance	0.032	0.01	2.43	0.015
Indirect effects					
SLA (cm2/gm)	Soil resources	4.950	0.045	110.000	0.000
Height	Soil resources	4.880	0.050	107.644	0.000
MPD	Soil resources	0.189	0.011	17.467	0.000
Leaf Production	Soil resources	5.073	0.047	106.957	0.000
c3-plants abundance	Soil resources	0.065	0.01	2.45	0.11
Height (cm)	Aridity Index	-0.339	0.037	-9.2	0.000
SLA	Aridity Index	-0.134	0.053	-2.515	0.012
leaf Production	Aridity Index	-0.163	0.133	-1.23	0.0219
MPD	Aridity Index	-0.02	0	-4.561	0.000
C3-plants abundance	Aridity Index	0.121	0.011	2.34	0.000
Soil resources	Aridity Index	0.008	0.001	15.995	0

### Indirect effects of climatic aridity and soil resources on SCBD

Climatic aridity and soil resources were the primary indirect influencers on SCBDeff. Climatic aridity had a significant negative effect on SLA, which was subsequently associated with an increase in SCBDeff (β = -0.034, p = 0.001). This finding suggests that higher aridity values correlate with lower SLA (β = -0.134, p = 0.012), indirectly contributing to an increase in SCBDeff. Furthermore, climatic aridity exerted negative effects on plant height and leaf production (β = -0.339, -0.163; p = 0.001, 0.012), which in turn was associated with a reduction in SCBDeff, indicating that elevated aridity leads to lower plant height and leaf production, indirectly decreasing SCBDeff.

Similarly, soil resources significantly positively affected height, SLA, and leaf production, which indirectly influenced SCBDeff. Higher soil resource availability was linked to increases in plant height and leaf production, indirectly raising SCBDeff. However, higher SLA in response to soil resources indirectly negatively affected SCBDeff. Additionally, the interaction between aridity and soil resources significantly positively affected SCBDeff (β = 0.008, p = 0.001), indicating that the combined influence of aridity and soil resources contributes positively to SCBDeff.

## Discussion

The study provides useful insights into the complicated mechanisms driving SCBDeff by analyzing the interconnections of native species characteristics, climate, and soil resources. We found that the SCBDeff is regulated by soil resources and climate aridity, with species traits functioning as significant modulators (Grace and Pugesek, 1997; Díaz et al., 2016). This indicates that the distribution of native plant species and their contribution to beta diversity is strongly influenced by the availability of water resources, as measured by the aridity index and soil water content (Fick and Hijmans, 2017; Trabucco and Zomer, 2019). These findings underscore the role of environmental factors in structuring plant communities, with implications for understanding ecosystem stability and resilience in arid regions, especially as climate change intensifies water scarcity (Allen et al., 2010; IPCC, 2014). The positive direct effect of climatic aridity on SCBDeff shows that higher aridity levels are linked to a greater contribution of certain studied species to beta diversity, such as *Achillea fragrantissima* and *Adiantum capillus-veneris*. *Achillea fragrantissima* is a drought-tolerant shrub commonly found in arid and semi-arid environments, thriving in rocky and sandy soils with minimal water availability. In contrast, *Adiantum capillus-veneris* is a fern species typically associated with moist, shaded microhabitats such as the edges of springs and damp rock crevices. Despite their contrasting habitat preferences, both species coexist within transitional zones where microclimatic variations create niches that support their growth, reflecting the diverse ecological conditions influencing beta diversity in arid landscapes. This suggests that certain native plant species in the study may flourish and predominate in more arid environments (Maestre et al., 2015).

Additionally, beta diversity encompasses both spatial and temporal dimensions. Climate change, by driving temporal turnover, may further influence the interplay between biotic and abiotic factors and SCBDeff (Harrison et al., 2015; Lenoir et al., 2020). Thus, shifts in beta diversity over time could alter the balance between these factors, affecting the overall ecosystem dynamics. Species features, including the mean pairwise phylogenetic distance (MPD), the relative abundance of C3 plants, leaf production, specific leaf area (SLA), and plant height, further regulate this positive effect (Webb et al., 2002; Swenson, 2011). These traits enable species to adapt to arid conditions, contributing more to beta diversity. For example, species with higher specific leaf area (SLA) had a lower contribution to SCBDeff, likely due to their water-conservative strategies (Chaturvedi et al., 2021; Wright et al., 2004).

In comparison to previous studies, this research emphasizes the importance of climatic aridity in shaping beta diversity (Dhirendra et al., 2022; Dietrich et al., 2021; Hoffmann et al., 2019; Götzenberger et al., 2012; Kraft et al., 2011). The positive relationship between SCBDeff and the aridity index suggests that species contribute more to beta diversity in increasingly arid conditions. This aligns with studies showing that arid environments select for species with drought-adaptive traits, promoting higher species turnover and greater contributions to beta diversity (Liu et al., 2019; Panja et al., 2022; Cornwell and Ackerly, 2009). Such conditions likely favor species with efficient water-use strategies, leading to a high turnover of species adapted to dry conditions. This turnover contributes significantly to beta diversity, enhancing resilience in arid environments by supporting a diverse range of adaptive strategies (Schimper, 1903; de Bello et al., 2013).

The significant direct effects of certain species traits on SCBDeff, such as plant height, leaf production, and the relative abundance of C3 plants, regulate their crucial role in driving SCBDeff. This observation is consistent with other research that has highlighted the significance of functional traits in determining species dominance and their contributions to beta diversity (Pardo, 2021; Peya, 2018; Westoby et al., 2002; Díaz et al., 2016). The negative direct effect of specific leaf area (SLA) on species contribution to beta diversity suggests that species with lower SLA values typically follow conservative water-use strategies, contributing less to beta diversity. This finding aligns with previous research emphasizing the role of water-use efficiency traits in arid ecosystems, where efficient resource utilization can provide a competitive advantage (Wright et al., 2005; Reich, 2014; Liu et al., 2022; Carvajal et al., 2019). However, with increasing aridity, the relationship between SLA variance and ecosystem stability may shift from positive to negative (García-Palacios et al., 2018). Species with low SLA values may be favored in drylands, as their conservative water-use strategies help maintain biomass stability (Díaz et al., 2006; Shipley et al., 2006). However, communities with high SLA variance may see shifts toward more competitive, drought-avoiding species, leading to greater variability in biomass and ecosystem processes (Lohbeck et al., 2015). Under these harsh conditions, communities with high SLA variance may indicate the replacement of stress-tolerant evergreen species by competitive, summer-deciduous plants that avoid drought through leaf shedding (Grime, 2006; Poorter et al., 2009), leading to increased variability in plant biomass over time.

The positive direct effect of mean pairwise phylogenetic distance (MPD) on SCBDeff highlights the crucial role of phylogenetic diversity in shaping species’ contributions to beta diversity. This finding indicates that distantly related species with greater evolutionary divergence tend to contribute more significantly to beta diversity than closely related species (Webb et al., 2002; Cavender-Bares et al., 2009). In the context of the study area vegetation, which includes desert and shrubland ecosystems, this effect is particularly pronounced. These ecosystems are characterized by harsh environmental conditions, where phylogenetic diversity among plant species may play a pivotal role in enabling community resilience and functional differentiation (Swenson, 2011; Graham and Fine, 2008). As species diverge through evolutionary history, they accumulate distinct traits and ecological roles that promote functional differentiation between communities (Hardy and Senterre, 2007). This process, known as niche differentiation, increases ecological differences across communities and subsequently enhances beta diversity. Cadotte (2017) emphasized that greater phylogenetic diversity increases the likelihood of complementary resource use, strengthening ecological differences between communities and driving higher beta diversity (Cadotte et al., 2009).

Phylogenetic diversity can also lead to phylogenetic over-dispersion, where distantly related species co-occur due to divergent functional traits that reduce competition and allow coexistence. This over-dispersion results in communities with higher functional differentiation, further contributing to beta diversity (Tucker and Cadotte, 2013; Ding et al., 2021; Montaño-Centellas et al., 2020; Zhao et al., 2022). Additionally, evolutionary history significantly drives ecosystem processes, as distantly related species exhibit a broader range of functional traits. Communities with high phylogenetic diversity are more likely to support varied ecosystem functions, such as nutrient cycling and productivity, due to the wide range of functional traits represented. This enhanced beta diversity could strengthen ecosystem resilience by allowing functional compensation during environmental changes. Communities with greater phylogenetic diversity are more likely to differ in ecosystem functions such as productivity, nutrient cycling, and resilience to environmental changes, which further enhances beta diversity (Flynn et al., 2011).

Alternatively, environmental filtering can lead to phylogenetic clustering, where harsh environmental conditions select closely related species with similar functional traits, thereby reducing beta diversity (Webb et al., 2002; Cavender-Bares et al., 2009; Swenson, 2013). However, in ecosystems with weaker environmental filtering, distantly related species with different adaptations colonize distinct environments, promoting higher beta diversity through greater community turnover (Helmus et al., 2007; Violle et al., 2011; Pavoine and Bonsall, 2011). Overall, the positive association between MPD and SCBDeff underscores the importance of considering evolutionary history in biodiversity assessments (Cadotte et al., 2008; Tucker et al., 2017). Distantly related species contribute more to beta diversity because their evolutionary divergence translates into greater functional differentiation and niche partitioning, which supports ecosystem functioning across different environments (Kraft et al., 2007; Mouquet et al., 2012; Gerhold et al., 2015). Incorporating phylogenetic diversity in conservation planning may help preserve evolutionary distinctiveness and ensure stable ecosystem functions in the face of environmental changes, particularly in arid regions (Faith, 1992; Srivastava et al., 2012; Winter et al., 2013).

The negative indirect effects of climatic aridity and soil resources on SCBDeff through specific leaf area, plant height, and leaf production highlight the complex influence of environmental factors on plant traits and community structure, indirectly affecting beta diversity (Diaz et al., 2007; Wright et al., 2005; Reich, 2014). Water scarcity due to climatic aridity can shape plant communities by promoting species with traits adapted to conservative water-use strategies, which may reduce their contributions to beta diversity (Grime, 2006; Cornwell and Ackerly, 2009; Liu et al., 2019). Similarly, while nutrient-rich soils generally support the growth of taller plants with numerous leaves, which positively influences beta diversity, they may also promote species with lower water-use efficiency (Maire et al., 2015; Craine et al., 2013). This could lead to a decrease in contributions to beta diversity due to the dominance of such species in resource-rich environments (Chapin et al., 2000; Swenson et al., 2012). Future research could further investigate these interactions by exploring additional environmental factors, such as temperature fluctuations and anthropogenic impacts, and examining SCBDeff across different ecosystems over time (Thuiller et al., 2011; Newbold et al., 2015). Such studies would provide valuable insights into how trait-environment interactions shape biodiversity patterns and ecosystem resilience in changing climates (Lavorel and Garnier, 2002; McGill et al., 2006).

## Conclusion

The results of this study highlight the complex interactions between species traits, climate aridity, and soil resources in shaping species contributions to beta diversity (SCBDeff). Climatic aridity emerged as a significant driver of SCBDeff, particularly in species with traits such as greater phylogenetic distance, taller plant height, higher leaf production, and a relative abundance of C3 plants. These traits enable species to adapt to arid conditions, contributing more to beta diversity. On the other hand, species with higher specific leaf area (SLA) had a lower contribution to SCBDeff, likely due to their water-conservative strategies. Phylogenetic diversity also played a pivotal role, with distantly related species contributing more to beta diversity due to functional differentiation and niche partitioning. Furthermore, the complex indirect effects of climatic aridity and soil resources on SCBDeff, mediated by species traits, emphasize how environmental factors shape plant communities and their contribution to ecosystem diversity. These results provide valuable insights into the mechanisms driving beta diversity, suggesting that species adapted to arid environments and with specific traits may play a pivotal role in maintaining ecosystem function and stability. The study findings highlight the importance of preserving species with key functional and phylogenetic traits, particularly in arid landscapes, where diversity is essential to sustaining ecosystem health. Understanding these interactions is essential for developing conservation strategies, particularly in arid and resource-limited ecosystems in the face of climate change, where monitoring the functional and phylogenetic diversity will be key to enhancing ecosystem stability. Future research should aim to explore the effects of additional environmental stressors, such as temperature extremes and habitat fragmentation, and assess these interactions over time. Such studies will provide essential data to guide conservation actions for ecosystem resilience in changing climates.

## Study limitations

Despite the valuable insights provided by this study, several limitations should be considered when interpreting the findings. Firstly, the study was conducted within a specific region (the Saint Katherine Protectorate in Egypt), which may limit the generalizability of the results to other arid regions with different environmental conditions. Additionally, the reliance on a single set of environmental variables (climate and soil resources) and plant traits, while comprehensive, may not fully capture the complex interactions that influence beta diversity in other ecosystems. The study’s focus on a limited number of plant traits, such as height, specific leaf area (SLA), and leaf number, may not account for the full spectrum of functional traits that could influence species contributions to beta diversity, potentially oversimplifying the role of functional diversity. Furthermore, although the study used advanced statistical techniques such as structural equation modeling (SEM) and beta regression to explore the relationships between environmental factors and SCBD, the complexity of these models may lead to issues with model fit or overfitting, especially given the limited sample size (84 plots). Lastly, while phylogenetic information was incorporated, the use of gene sequences from closely related species for some species may introduce uncertainties in phylogenetic relationships, potentially affecting the accuracy of the phylogenetic analyses. These limitations highlight the need for further studies across broader scales and with more diverse datasets to refine the understanding of beta diversity dynamics in arid ecosystems.

## Data Availability

The raw data supporting the conclusions of this article will be made available by the authors, without undue reservation.
