# Supplementary material for: Shaping beta diversity in arid landscape through native plant species contributions: synergy of climate, soil, and species traits
Source: Front Plant Sci. 2025 Mar 10;16:1521596. doi: 10.3389/fpls.2025.1521596 (PMC11950964; doi:10.3389/fpls.2025.1521596)
Supplement: Supplementary file 1 [file DataSheet1.pdf]

Supplementary Data to:

El-Barougy RF, Bersier L-F, Gray SM, El-Keblawy A, Galal T, Ullah F, Elgamal IA and Dakhil MA (2025) Shaping beta diversity in arid landscape through native plant species contributions: synergy of climate, soil, and species traits. Front. Plant Sci. 16:1521596.

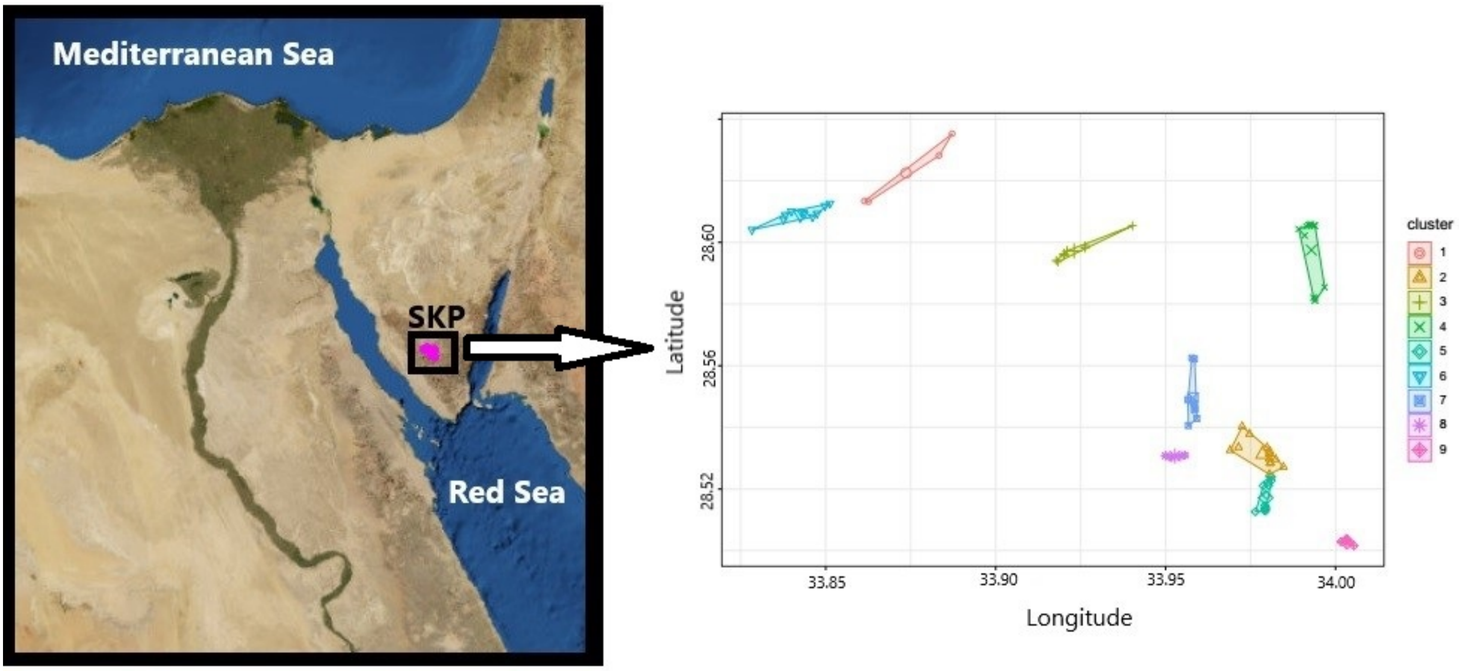

Fig. S1. Location of Saint Katherine Protectorate (SKP) the study area and the clusters of study plots (84 plots) on which SCBD was calculated.

Species Identity	**SCBD**
Achillea_fragrantissim	0.01012341
Adiantum_capillus_veneris	0.016300487
Alkanna_orientalis	0.051561106
Anarrhinum_pubescens	0.015897648
Anchusa_milleri	0.005510532
Arenaria_deflexa	0.001302489
Atraphaxis_spinosa	0.004315528
Ballota_kaiseri	0.009519352
Ballota_undulata	0.027216612
Blepharis_attenuata	0.007379031
Bufonia_multiceps	0.044977009
Capparis_sinaica	0.005372769
Caylusea_hexagyna	0.002865477
Chenopodium_album	0.002262219
Chiliadenus_montanus	0.030108432
Colutea_istria	0.000754073
Conyxa_aegyptiaca	0.003438572
Conyza_bonariensis	0.002249076
Cotoneaster_orbicularis	0.004796025
Crataegus_x_sinaica	0.006331071
Deverra_triradiata	0.006758535
Diplotaxis_harra	0.002865477
Echinops_glaberrimus	0.029631678
Ephedra_sp	0.005687294
Fagonia_arabica_arabica	0.002477432
Fagonia_mollis	0.049166013
Ficus_carica	0.000477579
Ficus_palmata	0.000795966
Galium_setaceum	0.002865477
Heliotropium_arbainense	0.000955159
Hyoscyamus_boveanus	0.023671772
Hypericum_sinaicum	0.012454254
Juncus_rigidus	0.007540729
Launaea_capitata	0.002729026
Launaea_nudicaulis	0.003306319
Lavandula_coronopifolia	0.007305889
Malva_neglecta	0.002262219
Matthiola_arabica	0.011866351
Matthiola_longipetala	0.002834134
Mentha_longifolia	0.030789909
Nepeta_septemcrenata	0.021572015

Species Identity	**SCBD**
Nicotiana_glauca	0.000573095
Origanum_syriacum	0.063976785
Peganum_harmala	0.004629726
Phlomis_aurea	0.085286472
Plantago_sinaica	0.002620832
Primula_boveana	0.077696794
Pterocephalus_sanctus	0.007026238
Retama_raetam	0.001102106
Rosa_arabica	0.054150262
Rumex_vesicarius	0.004249391
Salvia_multicaulis	0.00527851
Scrophularia_libanotica	0.001432738
Scrophularia_xanthogolssa	0.009282997
Seriphidium_herba_album	0.027870799
Silene_leucophylla	0.014178026
Silene_schimperiana	0.037844028
Solanum_nigrum	0.002865477
Stachys_aegyptiaca	0.018230195
Stipa_sp	0.015508379
Tanacetum_sinaicum	0.028634143
Teucrium_polium	0.007436307
Thymus_decussatus	0.001364513
Umbilicus_horizontalis	0.010728106
Verbascum_decaisneanum	0.007163692
Verbascum_sinaiticum	0.019681092
Zilla_spinosa	0.010895151

Table S1. Shows the list of studied native species and their SCBD values

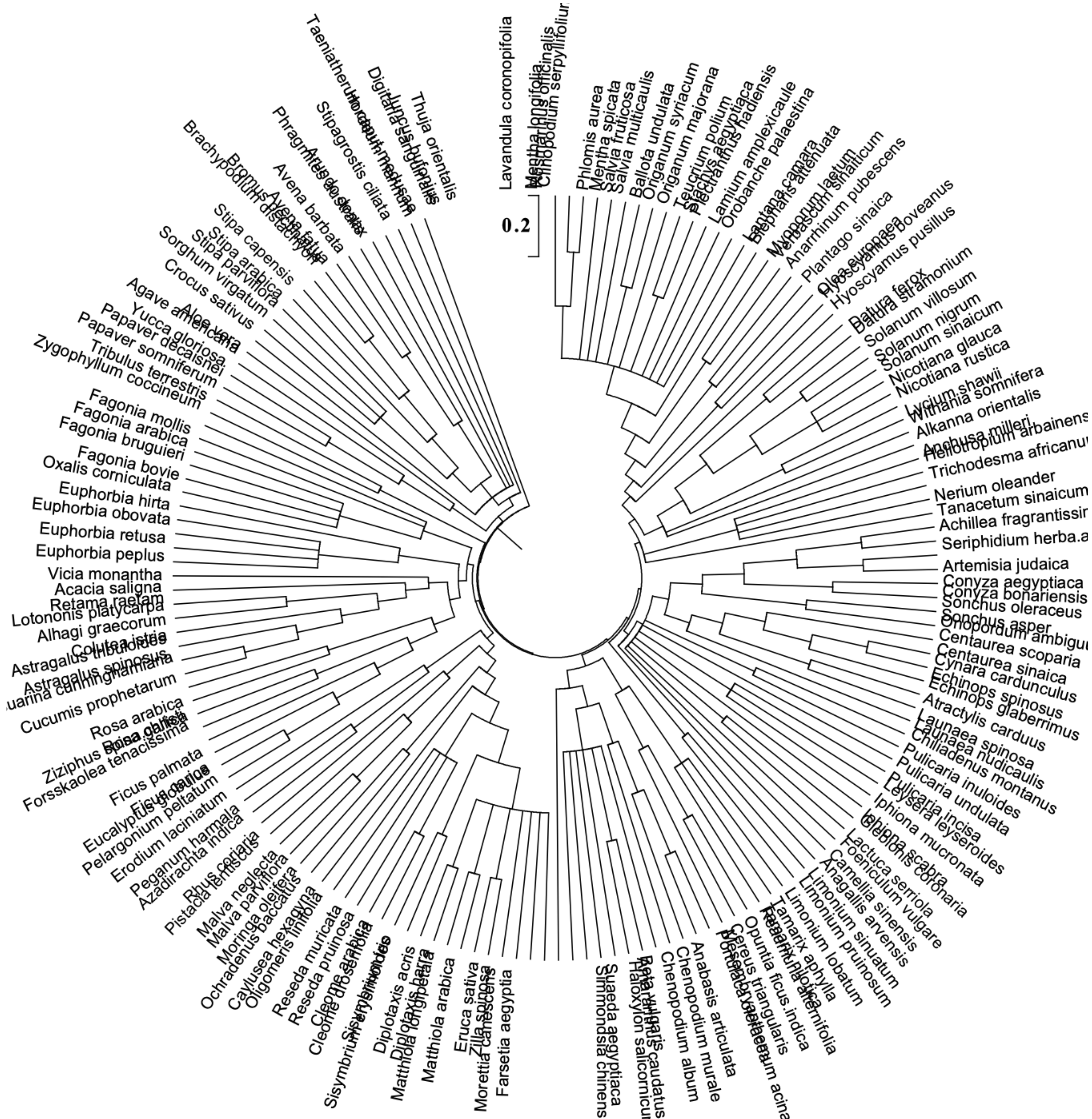

Fig. S2. Rooted ultrametric phylogenetic tree including the pool of 130 native species and 33 alien species collected from 83 pairs of invaded and non-invaded plots in Saint Katherine Protectorate. This tree was created by R.F.El-Barougy using PhyML-3.0 software ,version 3.0
